# GRK6 regulates ROS response and maintains hematopoietic stem cell self-renewal

**DOI:** 10.1038/cddis.2016.377

**Published:** 2016-11-24

**Authors:** Qiumin Le, Wenqing Yao, Yuejun Chen, Biao Yan, Cao Liu, Man Yuan, Yuqing Zhou, Lan Ma

**Affiliations:** 1 The State Key Laboratory of Medical Neurobiology, School of Basic Medical Sciences, the Institutes of Brain Science, and the Collaborative Innovation Center for Brain Science, Fudan University, Shanghai, China

## Abstract

G protein-coupled receptor kinases (GRKs) are critically involved in immune response through regulation of cytokine receptors in mature leukocytes, but their role in hematopoiesis is largely unknown. Here, we demonstrate that GRK6 knockout (GRK6^−/−^) mice exhibit lymphocytopenia, loss of the hematopoietic stem cell (HSC) and multiple progenitor populations. GRK6 deficiency leads to compromised lymphoid differentiation, largely owing to the impairment of HSC self-renewal. Transcriptome and proteomic analysis suggest that GRK6 is involved in reactive oxygen species signaling. GRK6 could interact with DNA-PKcs (DNA-dependent protein kinase, catalytic subunit) and regulate its phosphorylation. Moreover, reactive oxygen species scavenger *α*-lipoic acid administration could partially rescue the loss of HSC in GRK6^−/−^ mice. Our work demonstrates the importance of GRK6 in regulation of HSC self-renewal and reveals its potential role in participation of stress response.

G-protein-coupled receptor kinases (GRKs) are kinases that phosphorylate and desensitize agonist-bound G protein-coupled receptors (GPCRs).^1^ To date, GRKs have been shown to have critical regulatory roles in the neuronal,^2, 3^ cardiac^4^ and immune systems,^5, 6^ via GPCR-dependent and GPCR-independent mechanisms. Accumulating evidence implicates the importance of GRKs in regulating embryonic formation and development of key organs, including heart and brain. GRK2 knockout in mice are embryonic lethal due to marked cardiac abnormalities.^7, 8^ GRK2 mediates Smoothened-Hedgehog signaling desensitization,^9^ which regulates neural tube formation and muscle development in zebrafish and mice.^10^ Our earlier studies showed that GRK2 regulates cyclin B-dependent transcription, and downregulation of GRK2 in zebrafish embryos results in developmental early arrest and abnormal eye, midbrain and blood island formation.^11^ Recently we showed that GRK5 regulates neuronal morphogenesis and memory formation via a GPCR-independent mechanism.^12^

GRKs, especially GRK6, are highly expressed in vertebrate immune organs and peripheral blood cells.^13, 14^ GRK6 knockout (GRK6^−/−^) mice show increased severity of acute inflammatory arthritis^15^ and colitis^16^ because of enhanced granulocyte chemotaxis, and develop autoimmune diseases due to impaired macrophage engulfment.^17^ GRK6 regulates chemotaxis through SDF/CXCLs-CXCR4,^18, 19^ leukotriene B4-induced CGRP receptor^20^ and BLT receptor^21^ activation. Moreover, It has been reported that the expression and activity of GRK6 change during differentiation of the promyelocytic cell line HL-60,^22^ suggesting the potential involvement of GRK6 in earlier leukocyte development. However, the role of GRK6 in the development of blood lineages, from hematopoietic stem cells (HSCs) to differentiated immunoreactive leukocytes, is unclear.

The balance of cellular quiescence and proliferation of HSCs is critical for the preservation of their capacity for self-renewal and differentiation. In this study, we demonstrate that GRK6 regulates of self-renewal and lymphoid differentiation of HSCs, and underscore its underlying importance in the maintenance of reactive oxygen species (ROS) homeostasis.

## Results

### GRK6 knockout leads to lymphocytopenia

**Figure 1:**
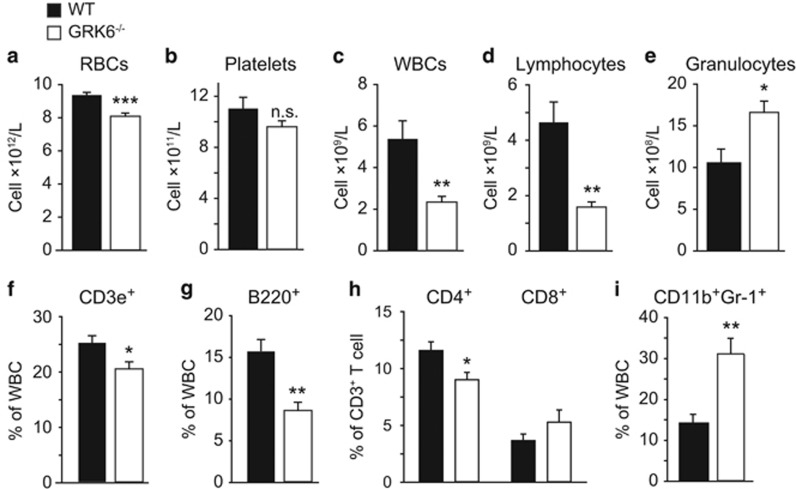
GRK6 knockout leads to lymphocytopenia. (**a–e**) Absolute number per liter of RBCs (**a**), platelets (**b**), WBCs (**c**), lymphocytes (**d**) and granulocytes (**e**) and (**f**–**i**) percentage of peripheral CD3e^+^ T cells (**f**), B220^+^ B cells (**g**), CD4 and CD8 single positive populations (**h**), CD11b^+^ Gr-1^+^ granulocytes (**i**) in total WBCs of 8–12 weeks old WT and GRK6^−/−^ mice (*n*=5–6 each). *P<0.05, **P<0.01, ***P<0.001 by two-tailed, unpaired Student's *t*-test. Data are expressed as mean±S.E.M.

To investigate the potential role of GRK6 in steady-state hematopoiesis, we first analyzed peripheral blood of 8–12 weeks old wild-type (WT) and GRK6^−/−^ mice. Compared with WT littermates, GRK6^−/−^ mice showed prominent decrease in lymphocyte number, and a slight reduction in red blood cells (RBCs) and platelets (Figures 1a–d). T- and B-cell percentages in GRK6^−/−^ mice both dropped, which further indicates lymphoid deficiency (Figures 1f and g). The CD4^+^ population of T cells was smaller than that of WT, suggesting immunosuppression (Figure 1h), but the total granulocyte number was slightly increased (Figure 1e), consistent with elevated CD11b^+^Gr-1^+^ granulocyte population (Figure 1i). The skewed peripheral blood constitution caused by GRK6 ablation suggests that it may participate in regulation of hematopoiesis and lymphoid differentiation.

### GRK6 knockout reduces HSC and progenitor populations

**Figure 2:**
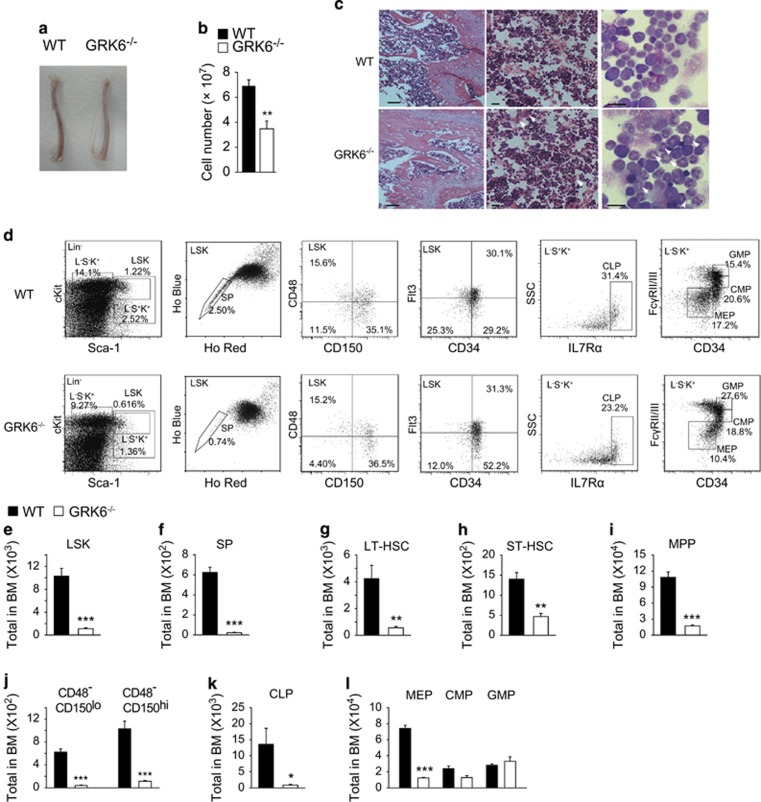
GRK6 knockout reduces BM HSC and progenitor populations. (**a** and **b**) Representative photograph of WT and GRK6^−/−^ tibia (**a**) and statistics of total cell number from two femurs and two tibiae of mice (*n*=6–7 mice each group) (**b**). (**c**) Femoral sections were subjected to HE staining (left, 40 × original magnification and middle, 400 × original magnification) and bone marrow smears were subjected to Wright-Giemsa's staining (right, 1000 × original magnification). Arrows indicate abnormal cells. Bars represent 500 *μ*m (left) or 20 *μ*m (middle and right). (**d**–**l**) Representative flow cytometric data (**d**) and (**e**–**j**) statistics of bone marrow LSK (Lin^−^Sca-1^+^cKit^hi^) (**e**), SP (LSKHoechst 33342^lo^) (**f**), LT-HSC (LSKFlt3^−^CD34^−^) (**g**), ST-HSC (LSKFlt3^−^CD34^+^) (**h**) and MPP (LSKFlt3^+^CD34^+^) (**i**), lymphoid competent HSC (LSKCD48^−^CD150^lo^) and myeloid competent HSC (LSKCD48^−^CD150^hi^) (**j**), CLP (Lin^−^Sca-1^+^cKit^+^IL7R*α*^+^Fc*γ*RII/III^−^) (**k**), CMP (Lin^−^Sca-1^−^cKit^+^Fc*γ*RII/III^lo^CD34^+^), GMP (Lin^−^Sca-1^−^cKit^+^Fc*γ*RII/III^hi^CD34^hi^) and MEP (Lin^−^Sca-1^−^cKit^+^Fc*γ*RII/III^lo^CD34^−^) (**l**) populations of 8–12 weeks old WT and GRK6^−/−^ mice (*n*=4–10 each). *P<0.05, **P<0.01, ***P<0.001 by two-tailed, unpaired Student's *t*-test. Data are expressed as mean±S.E.M. CMP, common myeloid progenitors; GMP, granulocyte/monocyte progenitors; LT-HSC, long-term HSC; MEP, megakaryocyte/erythrocyte progenitors; MPP, multi-potent progenitors; ST-HSC, short-term HSC

Dissected GRK6^−/−^ femurs and tibiae were paler, with fewer cell number than age-matched WT, suggesting bone marrow suppression (Figures 2a and b). Femoral H&E staining showed fewer cells in the marrow cavity (Figure 2c, left), markedly decreased RBCs, more early erythroid elements (Figure 2c, middle, arrowhead), while Wright-Giemsa revealed a notable increase in ring-like nuclei of immature myeloid cells in bone marrow smears (Figure 2c, right, white arrowhead) of GRK6^−/−^ bone marrow (BM). These results indicate that GRK6 knockout leads to distorted hematopoietic cell maturation.

We then examined hematopoietic stem cell and progenitor populations to find out the fractions affected by GRK6 ablation. The results showed decreased HSC populations, LSK (Lin^−^Sca-1^+^cKit^hi^), and side population (Hoechst 33342^lo^LSK) in GRK6^−/−^ BM (Figures 2d–f). The HSC sub-populations, long-term HSC, and short-term HSC, as well as multi-potent progenitor in GRK6^−/−^ BM dropped dramatically (Figures 2d and g–i). Loss of lymphoid competent HSC^23, 24^ and lower common lymphoid progenitor (CLP) population were observed (Figures 2d, j and k), indicating lymphoid hematopoiesis deficiency in GRK6^−/−^ BM. Common myeloid progenitors and megakaryocyte/erythrocyte progenitors in GRK6^−/−^ BM were also reduced, while the number of granulocyte/monocyte progenitors did not change (Figures 2d and l). The loss of these progenitor populations is consistent with decreased peripheral RBCs and lymphocytes, indicating the importance of GRK6 in HSC maintenance and differentiation.

### GRK6 is essential for HSC self-renewal

**Figure 3:**
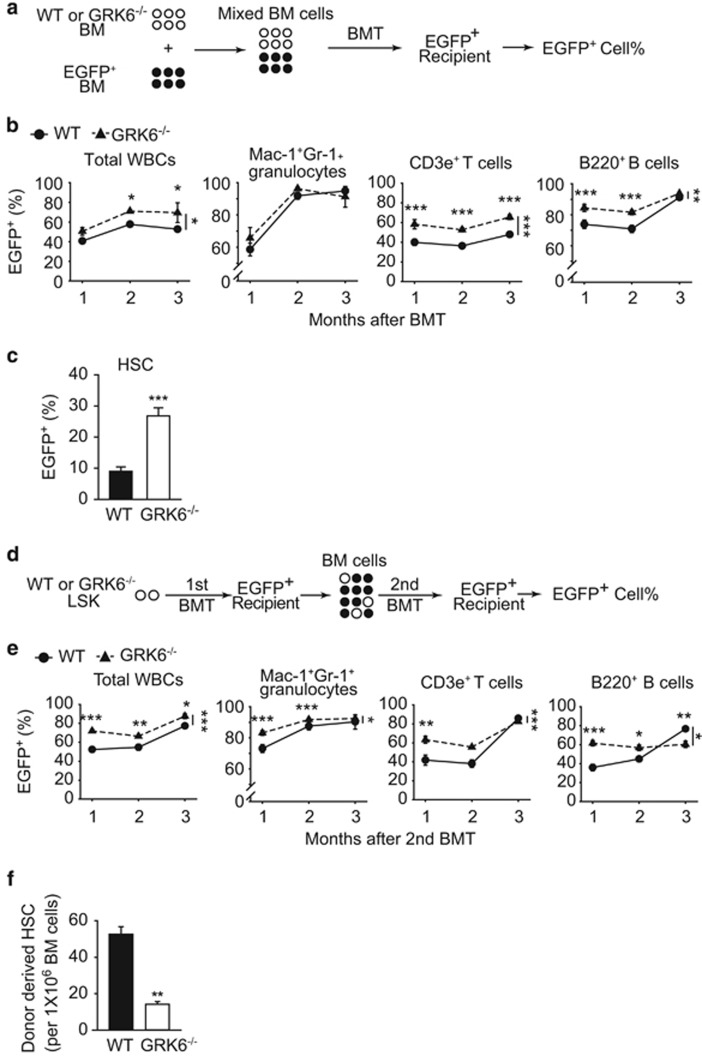
GRK6 is essential for self-renewal of HSCs. (**a**–**c**) Competitive BM transplantation assay. (**a**) A 1:1 mixture of BM cells from EGFP^+^ and WT or GRK6^−/−^ mice was transplanted into a lethally-irradiated EGFP^+^ recipient mouse. (**b**) Reconstitution after transplantation was presented as EGFP^+^ percentage in peripheral WBC, granulocyte, T-cell and B-cell populations (recipient, *n*=6; donor, *n*=3 per genotype). Two-way ANOVA with Bonferroni *post hoc*, *P<0.05, **P<0.01, ***P<0.001 between data points (on top) or genotypes (on the right). (**c**) EGFP^+^ percentage in HSC populations 3 months post transplantation. **P<0.001 by two-tailed, unpaired Student's *t*-test. (**d**–**f**) Serial transplantation assay. (**d**) 1000 sorted WT or GRK6^−/−^ LSK cells were transplanted into an lethally-irradiated EGFP^+^ recipient mouse (the primary transplantation), and the BM cells from the primary recipient were then transplanted into another EGFP^+^ recipient (the secondary transplantation). (**e**) EGFP^+^ percentage of peripheral blood cells after the secondary transplantation (recipient, *n*=4–6; donor, three per genotype). Two-way ANOVA with Bonferroni *post hoc*, *P<0.05, **P<0.01, ***P<0.001 between data points (on top) or genotypes (on the right). (**f**) HSC number 3 months after the secondary transplantations (recipient *n*=6 each, donors *n* = 4 each). **P<0.01 by two-tailed, unpaired Student's *t*-test. All data are expressed as mean±S.E.M.

To investigate the function of GRK6 in hematopoiesis, WT or GRK6^−/−^ BM was transplanted along with EGFP^+^ BM as competitor (Figure 3a). The results show that peripheral reconstitution of granulocytes was comparable (Figure 3b). However, higher percentage of EGFP^+^ cells was observed in total peripheral reconstitution, as well as T cells and B cells of GRK6^−/−^ BM recipients than that of WT BM recipients (Figure 3b). Moreover, higher percentage of EGFP^+^ HSCs was observed in bone marrow of GRK6^−/−^ recipients than those of WT recipients (Figure 3c), indicating lower ability of GRK6^−/−^ HSCs to reconstitute. These data suggest that GRK6 may regulate HSC maintenance.

Serial transplantation of sorted WT and GRK6^−/−^ LSKs was carried out to assess the potential role of GRK6 in HSC homeostasis (Figure 3d). To evaluate the reconstituting capability of WT and GRK6^−/−^ HSC, we quantified the recipient-derived residual GFP^+^ cells after serial transplantation. The secondary recipients of GRK6^−/−^ LSK cells exhibited higher EGFP^+^ percentage of WBCs, granulocytes, T cells and B cells than those received WT LSK cells (Figure 3e), and consistently, smaller GRK6^−/−^-derived HSC population (Figure 3f) was observed, indicating stem cell exhaustion. The above data indicate that GRK6 regulates HSC-intrinsic self-renewal.

### GRK6 regulates differentiation of hematopoietic progenitor cells

**Figure 4:**
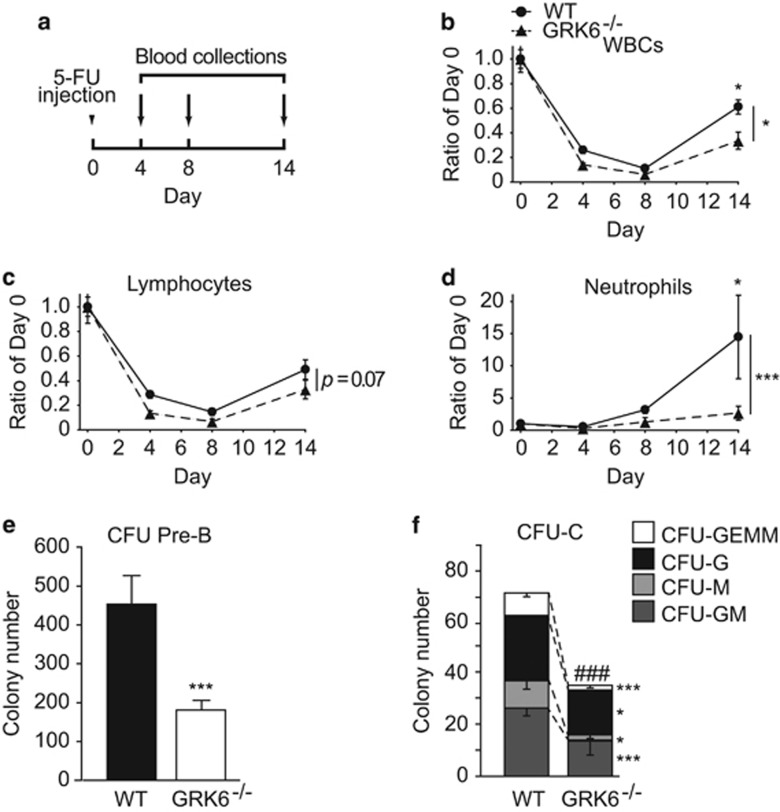
GRK6 regulates lymphoid differentiation. (**a**–**d**) Single-dose 5-FU assay for proliferation. (**a**) GRK6^−/−^ mice and WT littermates were intraperitoneally injected with a single dose of 5-FU on day 0 followed by serial samplings of peripheral blood at day 4, 8, 14 (*n*=3–7 each). Peripheral blood count of (**b**) WBCs, (**c**) lymphocytes and (**d**) neutrophils were determined with hematology analyzer and presented as percentage of original cell count. Two-way ANOVA with Bonferroni *post hoc*, *P<0.05, ***P<0.001, on the right, within genotype; on top, within each day. Data are expressed as mean±S.E.M. (**e**,**f**) Whole bone marrow culture to test clonogenic ability with (**e**) CFU-Pre-B (*n*=3 each with duplicate) or (**f**) CFU-C assay (*n*=5 each with duplicate). On top, ^###^*P*<0.001 of total colony number in (**f**); *P<0.05, ***P<0.001 WT *versus* GRK6^−/−^ of each population by two-tailed, unpaired Student's *t*-test. Data are expressed as mean±S.E.M. 5-FU, 5-Fluoruracil

Loss of lymphocytes in GRK6^−/−^ mice suggests that GRK6 may also participate in regulating differentiation. Serial peripheral blood sampling after a single 5-fluoruracil injection revealed slower rebound of number for WBCs, lymphocytes, and neutrophils in GRK6^−/−^ mice (Figure 4a and d). *In vitro* clonogenic experiments showed decreased pre-B colonies (CFU-Pre-B) (Figure 4e) and loss of granulocyte (CFU-G), macrophage (CFU-M) and multi-potential mixed colonies (CFU-GM and CFU-GEMM) (Figure 4f) in GRK6^−/−^ BM. The defect in lymphoid and myeloid repopulation and progenitor differentiation indicates that GRK6 regulates the differentiation of HSC.

### GRK6 regulates DNA-PKcs phosphorylation and ROS response

**Figure 5:**
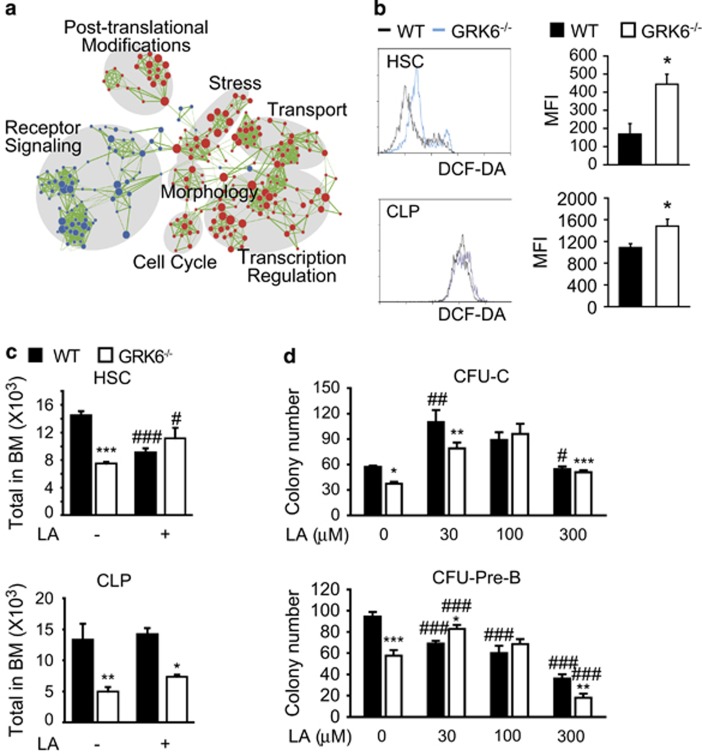
Oxidative stress is involved in hematopoietic deficiencies caused by GRK6 ablation. (**a**) Network analysis of over-represented pathways in RNA-seq. Each node represents a pathway, its size correlates with enrichment significance and edges represent gene overlaps between nodes. Network clusters are indicated by grey shadows. (**b**) Representative flow cytometric analysis and statistics of ROS level assessed by DCF-DA fluorescence (*n*=5, 3). *P<0.05 by two-tailed, unpaired Student's *t*-test. (**c**) WT and *GRK6*^−/−^ mice was randomized to i.p. injection of saline or LA (50 mg/kg) for two weeks (WT+Saline, WT+LA, GRK6^−/−^+Saline, GRK6^−/−^+LA, *n*=5, 4, 3, 3). HSC, two-way ANOVA, with Bonferroni *post hoc*, *P*=0.01, WT *versus* GRK6^−/−^, *P*=0.305, LA *versus* Saline. CLP, two-way ANOVA with Bonferroni *post hoc*, *P*=0.015, WT *versus* GRK6^−/−^; *P*=0.874, LA *versus* control; **P<0.01, ***P<0.001, within genotype; ^#^*P*<0.05, ^###^*P*<0.001, within treatment. (**d**) Methocellulose-based CFU-C and CFU-Pre-B assay at different LA dosage. CFU-C, two-way RM ANOVA with Bonferroni *post hoc*; *P*=0.107, WT *versus* GRK6^−/−^; *P*<0.001, LA dosage. CFU-Pre-B, two-way RM ANOVA with Bonferroni *post hoc*, *P*=0.091, WT *versus* GRK6^−/−^; *P*<0.001, LA dosage. *P<0.05, **P<0.01, ***P<0.001, Bonferroni *post hoc* within genotype; ^#^*P*<0.05, ^###^*P*<0.001, Bonferroni *post hoc* within treatment (versus LA dose 0). Data are expressed as mean±S.E.M.

Transcriptome analysis of HSC and CLP populations in WT and GRK6^−/−^ mice was carried out. RNA-seq result revealed that the expression levels of 475 genes in HSC and 1160 genes in CLP populations were changed significantly (log_2_ (fold)⩾1 or ⩽−1, *P*⩽0.05). We used gene set enrichment analysis to correlate expression alterations with hallmark gene sets and summarized relevant biological states. As shown in Supplementary Figure 1a, GRK6 knockout leads to most significant alterations in cMyc-regulated genes, ROS signaling (Supplementary Figure 1b), and DDR pathway etc., in both HSC and CLP populations. Enrichment map^25, 26^ analysis was used to sum up significantly altered pathways, which suggest that receptor signaling, transcription regulation, post-translational regulations, and notably stress and cell cycle related pathways were involved in GRK6-dependent mechanisms in hematopoiesis (Figure 5a).

We then asked if oxidative stress is involved in the phenotypic defects caused by GRK6 ablation. ROS is a major source of oxidative stress. DCF-DA staining showed that GRK6 ablation resulted in elevated ROS level (Figure 5b) in HSC and CLP populations. Moreover, DNA damage-induced *γ*H2AX phosphorylation was significantly aggravated in GRK6^−/−^ HSC (Supplementary Figures 2a and b). These data indicate that GRK6 may participate in cellular ROS cleavage and DNA damage repair.

*In vivo* and *in vitro* antioxidant treatment was utilized to see if increased ROS is causal. The results show that 50 mg/kg *α*-lipoic acid (LA) treatment significantly increased HSC count and CLP was slightly increased CLPs in GRK6 knockout mice (Figure 5c). In methocellulose-based cultures (Figure 5d), 100 *μ*M LA supplementation restored myeloid colony number of GRK6^−/−^ bone marrow, and 30 *μ*M LA was beneficial to growth of Pre-B colonies. Quantitative PCR analysis of HSC and CLP showed the expression of several genes that dominantly regulates stress response, namely *Sod2*, *Sod3*, *Nrf2* and *Cdkn1a* in HSC, and *Sod3* and *Cdkn1a* in CLP were significantly changed. (Supplementary Figure S1c).

In parallel, we investigated the effect of GRK6 knockdown in Jurkat cells. Lentivirus-based shRNA was designed to target common exons of GRK6 (Supplementary Figures 3a and b). GRK6 knockdown severely inhibited the growth of Jurkat cells (Supplementary Figure 3c), and the effect could be alleviated by 30 *μ*M LA supplementation (Supplementary Figure 3d). As GFP introduced by lentivirus infection interferes with DCF-DA fluorescence, we measured Trolox-equivalent antioxidant capacity, which was down-regulated by GRK6 knockdown, and was slightly reversed by LA supplementation at 100 *μ*M (Supplementary Figures 3e), and coincides with *in vivo* observations. Collectively, ROS quenching could rescue loss of HSC number and myeloid clonogenic ability in GRK6-deficient bone marrow cells, and partially alleviate *in vitro* lymphoid differentiation and growth of lymphoid cell line. Taken together, these data suggest that excessive ROS level in GRK6^−/−^ hematopoietic stem and progenitor cells contributes to loss of HSC self-renewal ability.

**Figure 6:**
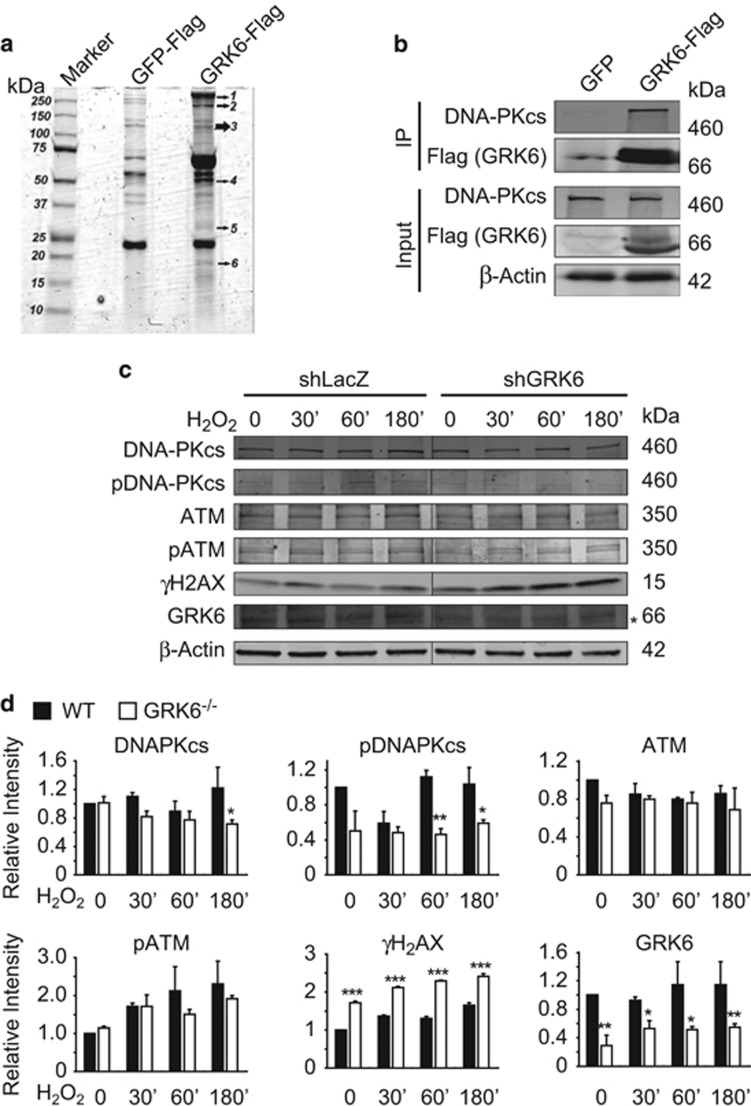
GRK6 regulates DNA-PKcs phosphorylation. (**a**) IP–MS of GRK6 in 231 cell line. Numbers indicate bands that were excised and subjected to mass spectrometry analysis. (**b**) GRK6 interacts with DNA-PKcs. HEK293T cells were transfected with FLAG-GRK6 or FLAG-GFP, and immunoprecipitated with anti-FLAG M2 beads. (**c**) H_2_O_2_ stimulation of Jurkat T cells infected with control (shLacZ) or GRK6 knockdown (shGRK6) lentivirus. Infected cells were serum starved for 8 h, treated with 200 *μ*M H_2_O_2_ and collected at 0, 30, 60, 180 min afterwards. *GRK5 band recognized by GRK4-6 antibody. (**d**) Densitymetric analysis of DNA-PKcs, pDNA-PKcs, ATM, pATM, *γ*H2AX and GRK6 (*n*=3). Expression level were normalized to *β*-ACTIN and shown as relative level to LacZ-0 min. Two-way ANOVA with Bonferroni *post hoc*; *P<0.05, **P<0.01, ***P<0.001, within genotype; ^#^*P*<0.05, ^###^*P*<0.001, Bonferroni *post hoc* within treatment (*versus* LA dose 0). Data are expressed as mean±S.E.M.

To gain insight into molecular mechanism by which GRK6 regulates stress-related response, we tried to identify GRK6 interacting proteins with immunoprecipitation and mass spectrometry (IP–MS) analysis (Figure 6a). Interestingly, besides known substrates such as HSP90AA1, HSP90AA2 and HSP90AB1, proteomic screening revealed association of GRK6 with members of phosphatidylinositol-3-kinase-related kinase (PIKK) family, including ATM, ATR, and DNA-PKcs. Especially, 220 peptide fragments from DNA-PKcs were detected. DNA-PKcs is known to mediate non-homologous end joining and lymphocyte-specific V(D)J recombination,^27^ and is recently reported as an ‘ROS sensor'.^28^ In response to H_2_O_2_ treatment (200 *μ*M), reduced phosphorylation of DNA-PKcs (pT2056), but not pATM (pS1981) was observed (Figure 5d). Moreover, DNA damage-induced *γ*H2AX phosphorylation was significantly aggravated in GRK6-deficient cells (Figures 6c and d). Taken together, the interaction between DNA-PKcs and GRK6 might be essential for DNA-PKcs phosphorylation, which mediates cellular protection against oxidative stress, and possibly participates in lymphoid differentiation.

## Discussion

GRKs have shown broad distribution in various tissues and act on a variety of substrates. GRK6 was known to critically control chemotaxis and autoimmune processes of mature blood cells. Here we report a novel aspect of GRK6 function in hematopoietic stem cell maintenance. We found that GRK6 ablation lead to pronounced lymphocytopenia, fewer HSCs, and smaller common lymphoid progenitor population. We also proved that GRK6 is essential to HSC self-renewal. Increased ROS level and DNA damage in *GRK6*^−/−^ HSC and CLP suggest involvement of GRK6 in maintaining redox homeostasis, and antioxidant treatment could at least partially rescue the loss of HSC and clonogenic ability of GRK6-deficient bone marrow. Our data suggest an indispensable role of GRK6 in regulating hematopoietic stem cell renewal.

Regulation of ROS level is critical in maintaining stem cell self-renewal and differentiation, as well as in treatment of stem cell associated diseases. High level of ROS has been long suggested detrimental.^29, 30, 31^ However, several studies have shown that low physiological level of ROS and other stressors operate as intracellular signaling molecules and promote stem cell renewal and differentiation.^32, 33, 34^ Redox regulation of HSC quiescence and self-renewal, lymphoid and myeloid balance,^34^ and T-cell differentiation^35^ have been reported in recent literatures.^36^ We found that GRK6-related redox disturbance could be rescued by a relatively low dose of *α*-lipoic acid (Figures 5, 6 and Supplementary Figure 2). Interestingly, in healthy mice, HSC count was unexpectedly reduced by *in vivo* LA administration (Figure 5c). Furthermore, in WT bone marrow culture, whereas CFU-C ability was improved by 30 *μ*M LA, LA supplementation seems destructive to Pre-B growth (Figure 5d) and growth of healthy Jurkat cells (Supplementary Figure 3d). These data implicate that differentiation of myeloid and lymphoid progenitors require distinct ROS level, and ROS seems essentially promotes lymphoid growth and differentiation. Furthermore, excessive antioxidants administration could be hazardous to normal objects, and should be administrated with caution.

In our IP–MS results, 220 peptides from DNA-PKcs were detected, in contrast to the few fragments found from ATM and ATR, the other 22 PIKK family members, indicating specific and strong protein association. DNA-PKcs mediates non-homologous end joining pathway and lymphocyte-specific V(D)J joining by recruiting its complex components to the sites of DNA double-strand break. DNA-PKcs knockout or Thr2609 cluster mutation leads to premature-aging and immunodeficiency due to blocked V(D)J rearrangement and DNA damage accumulation in mice.^37^ DNA-PKcs was also reported to be an ‘ROS sensor'. In response to ROS signal, DNA-PKcs undergoes phosphorylation at Thr2056 and subsequently phosphorylates p53 as well as other downstream transcription factors.^38^ Interestingly, although both HSC and CLP exhibit higher ROS level and altered cellular stress pathway in GRK6^−/−^ mice, they responded differentially to LA dose and treatment, and CLP number as well as lymphoid differentiation was not restored by ROS scavenger. It is likely that redox-independent functions of DNA-PKcs, like non-homologous end joining in lymphocytes, are involved. One thing to note is that GRK6 has many splice variants which differ in terms of their regulation by carboxyl-terminal post-translational modification.^39^ These variants exhibit differential subcellular and tissue distribution.^40^ The GRK6 knockout mice were generated through deletion of common exons 3–9. Therefore, the GRK6 ablation phenotype was derived from functional inactivation of all splice variants. Clarifying the dominant isoform of GRK6 in hematopoiesis would help to further the understanding of GRK6 function. Further mechanisms, like how GRK6 mediates phosphorylation of DNA-PKcs and whether it depends on kinase activity of GRK6, needs to be further elucidated.

Previous studies indicated that GRK6 participates in cytokine and chemokine-mediated immune responses. It was demonstrated that perturbed CXCR4 signaling leads to abnormalities of mature and immature hematopoiesis, while GRK6 negatively modulates ligand-induced CXCR4 desensitization. Thus enhanced CXCR4-mediated neutrophil chemotaxis and impaired responsiveness to G-CSF was observed in GRK6-deficient mice.^41^ In transcriptome analysis, we also identified alterations in receptor and ligand expression in this well-reported regulatory axis in GRK6-deficient HSC and CLP (Figure 5a). However, in spleen clonogenic assay, comparable clonogenic ability of GRK6^−/−^ and WT bone marrow cells was observed, indicating dispensable migration and reconstitution of hematopoietic progenitor cell activity in GRK6 ablation (Supplementary Figure 4). In combination with other data presented, we believe that the novel function of GRK6, in regulating DNA-PKcs phosphorylation and maintaining redox homeostasis, is a critical pathway that maintains HSC self-renewal and differentiation.

In conclusion, our study provides evidence that GRK6 is essential to the maintenance of self-renewal of HSC, and acts as a regulator of redox homeostasis. Given that GRK6 knockout mice are viable, and prominent growth arrest of lymphoid leukemia cell line was observed upon GRK6 knockdown, it might be feasible that GRK6 be used as a potential target for treatment of leukemia.

## Materials and Methods

### Animals

GRK6 knockout strain backcrossed to C57BL/6 background was kindly provided by RJ Lefkowitz and RT Premont (Duke University Medical Center, Durham, NC, USA). WT and GRK6 knockout (GRK6^−/−^) littermates were produced by GRK6 heterozygote crossing and genotyped by triplex PCR amplification using tail tip genomic DNA.^42^ Transgenic mice with ubiquitous somatic expression of enhanced green fluorescent protein (C57BL/6-Tg(CAG-EGFP)1Osb/J strain, hereafter referred to as EGFP mice)^43^ was purchased from Model Animal Research Center of Nanjing University and maintained by homozygote crossing. Mice were on a reversed 12h light/dark cycle with food and water available *ad libitum*. Mice used for experiments were 8–12 weeks old unless otherwise specified. All experiments were performed in accordance with the National Institutes of Health Guide for the Care and Use of Laboratory Animals, and approved by Animal Care and Use Committee of Shanghai Medical College, Fudan University.

### Hematology analysis

Peripheral blood was obtained by retro-orbital puncture into EDTA**·**K_2_-treated tubes. Complete blood counts were performed on Sysmex KX-21N or XS-800i hematology analyzers (Sysmex Corporation, Kobe, Japan).

### Flow cytometric analysis

BM cell suspension was prepared by flushing femurs and tibias with IMDM medium containing 2% fetal bovine serum (FBS). Red blood cells were lysed with ACK lysis buffer (0.15 M NH_4_Cl, 1 M KHCO_3_, 0.1 mM EDTA, pH 7.4) before immunostaining. Cell suspensions were filtered through a 40 *μ*m mesh, incubated with antibodies or dyes as indicated, and subjected to analysis or sorting on Moflo XDP (Beckman Coulter Inc., Brea, CA, USA), FACSAria III (BD Biosciences, San Jose, CA, USA) or Guava EasyCyte 8HT (Merck KGaA, Darmstadt, Germany). Raw data were analyzed with FlowJo (Version 7.6; FlowJo LLC, Ashland, OR, USA). The antibodies used, including CD3 (145-2c11), CD45R/B220 (RA3-6B2), CD11b (M1/70), Erythroid marker (Ter-119), Ly-6G (RB6-8C5), CD117/cKit (ACK2), Flt3 (A2F10), CD127 (A7R34), IgM (ll/41), Sca-1 (D7), CD16/32 (93), CD19 (ebio1D3), CD24 (M1/69), CD25 (PC61.5), CD34 (RAM34), CD43 (eBio2/60), CD44 (IM7), CD150 (mShad150) and CD48 (HM48-1), were from eBioscience (San Diego, CA, USA). CD4 (RM4-5) and CD8a (53-6.7) were from BD Biosciences. The total cell numbers were acquired by multiplying the total trypan blue negative cells enumerated with TC10 Automated Cell Counter (Bio-Rad Laboratories, Hercules, CA, USA) with the frequency of cells of the indicated populations within the PI (propidium iodide)-negative and FSC/SSC gates. For ROS measurement, cells were diluted to 5 × 10^6^ cells/ml, and incubated with 20 *μ*M 2′, 7′-Dichlorodihydrofluorescein diacetate (DCF-DA) (Enzo Life Sciences, Farmingdale, NY, USA) for exactly 30 min at 37 °C. Side population (SP) was stained by incubating 1 × 10^6^ cells/ml with 5 mg/ml Hoechst 33342 for 90 min at 37 °C.

### Bone marrow transplantation

EGFP mice were used as transplantation recipients. In competitive transplantation assay, BM cells from 8–12 weeks old WT or GRK6^−/−^ littermates were 1:1 mixed with EGFP^+^ BM cells and 1 × 10^6^ cells were intravenous injected into each EGFP recipient. In non-competitive serial transplantation assay, a mixture of 1000 LSK cells from WT or GRK6^−/−^ BM and 1 × 10^5^ EGFP^+^Sca-1^−^ cells from BM of EGFP mice were intravenously injected into each EGFP^+^ recipient. Three months later, BM cells from these recipients were used for secondary transplantation. The recipients were GFP^+^ transgenic mice and the donors were WT or GRK6^−/−^ mice which carry no GFP fluorescence. The recipient-derived residual GFP^+^ cells were quantified to evaluate the reconstitution capability of WT and GRK6^−/−^ HSCs. All recipient mice received 8.5 Gy ionizing radiation (IR). Transplantation was performed within 6 h post irradiation. EGFP^+^ percentage of each peripheral and bone marrow population was analyzed by flow cytometry as indicated.

### CFU-S assay

For CFU-S assay, 2 × 10^5^ BM cells from WT or GRK6^−/−^ mice were injected into lateral tail veins of 8.5 Gy-irradiated C57BL/6 recipient mice (*n*=4–6 each). On day 12, spleens were dissected and analyzed as described.

### Bone marrow clonogenic assay

BM cells were plated in duplicate in methocellulose-based IMDM medium supplemented with indicated cytokines (for CFU-C assay, 2 × 10^4^ cells with 50 ng/ml SCF, 10 ng/ml IL-2, and 10 ng/ml IL-6; for CFU-Pre-B assay, 5 × 10^4^ cells with 5 ng/ml SCF and 10ng/ml IL-7). In *α*-lipoic acid treatment experiments, 0, 30, 100, 300 *μ*M *α*-lipoic acid (LA) was added to the culture media. Cells were cultured at 37 °C in 5% CO_2_ for 6 days (CFU-Pre-B) or 10 days (CFU-C) before enumeration. Colony number was scored based on StemCell Technologies criteria.

### 5-Fluoruracil assay

5-Fluoruracil was administered intraperitoneally at 150 mg/kg on day 0. Retro-orbital bleeding was performed at indicated time and blood samples were assayed with a Sysmex XS-800i hematology analyzer.

### Mice *α*-Lipoic acid treatment

For *α*-lipoic acid treatment, eight-week old mice were given daily i.p. 50 mg/kg *α*-lipoic acid administration for two weeks, and mice were sacrificed 24 h. after the last injection.

### Immunofluorescence and histological microscopy

HSCs were sorted onto poly-l-lysine coated slides with and fixed with 4% paraformaldehyde for 15 min. Cells were permeablized with 0.3% Triton X-100, blocked with 1% FBS+0.1% BSA in PBS, incubated with *γ*H_2_AX antibody (JBW301) overnight at 4 °C and counterstained with DAPI (4,6 diamidino-2-phenylindole). Images were captured with LSM510 META confocal microscope (Carl Zeiss, Oberkochen, Germany). For each cell, z-stack of optical sections, 0.75 *μ*m each and 15 *μ*m in total thickness was performed and merged into one with Aim Image Browser (Carl Zeiss). Dots within DAPI positive area were enumerated with Image-Pro plus 6.0 (Media Cybernetics Inc., Rockville, MD, USA). At least 100 cells were calculated in each population. For H&E staining, femur from WT and GRK6^−/−^ mice were fixed in 4% paraformaldehyde, and decalcified in 14% EDTA, sectioned and stained with hematoxylin and eosin (H & E). Bone marrow smears were prepared and subjected to Wright-Giemsa staining. Images were captured with Olympus BX41 microscope.

### Plasmid construction

Plasmids encoding Flag-tagged human GRK6 splice variant 1 and GFP were constructed into pCDNA3.0. Construction of shRNA plasmids for human GRK6 was performed as described.^44^ Briefly, common coding sequence of human GRK6 mRNA sequence was analyzed by BLOCK-iT RNAi Designer and shRNAs were picked out, synthesized and constructed into FG12-hU6-shRNA vector. The shRNA lentivirus system was obtained from Dr. Gang Pei (Chinese Academy of Sciences, Shanghai, China). LacZ shRNA was used as a control. Sequences used are shGRK6, GCCTGTTATTTCGTGAGTTCT (sense); shLacZ, GCTAAATACTGGCAGGCGTTT (sense).

### Cell culture

Jurkat cell line was maintained in RPMI-1640 supplied with 10% fetal bovine serum (FBS, HyClone Laboratories Inc., South Logan, UT, USA). HEK293T cells were cultured in Dulbecco's modified Eagle's medium containing 10% FBS. MDA-MB-231 cells stably expressing GFP or GRK6-Flag were cultured in Leibovitz's L-15 plus 10% FBS. The cells were obtained from the lab of Gang Pei and tested for mycoplasma contamination prior to experiments.

### Generation and titration, and infection of lentivirus

HEK293T cells were seeded in 100 mm cell culture dishes at 3 × 10^6^ cells/dish, transfected with FG12 and packaging vectors, and the resulting supernatant was collected after 48 h. Titer were determined by 293T infection. The titer of lentivirus was ~10^7^ infectious units (IFU)/ml in a typical preparation and concentration. Jurkat cells were infected by spinoculation at MOI of 30 and cultured for at least 72 h before cellular assay.

### Immunoprecipitation followed by mass spectrometry

The experiment was performed as specified in Wu *et al.*^45^ Briefly, MDA-MB-231 cells were transfected with GRK6-Flag or GFP-Flag lentivirus, harvested and lysed (50 mM Tris, pH 7.5, 150 mM NaCl, 0.5% NP-40, 10% glycerol, 1 mM EDTA, 10 mM NaF, 10 *μ*g/ml aprotinin, 10 *μ*g/ml benzamidine, and 0.2 mM PMSF). The lysate was incubated with anti-FLAG M2 affinity gel and bound proteins were electrophoresed on a 4–20% gradient polyacrylamide gel and revealed by staining with Coomassie Brilliant Blue G250. Selected bands were excised from the gel and analyzed by mass spectrometry at the Shanghai Applied Protein Technology (Shanghai, China). MS/MS spectra were automatically searched against the non-redundant International Protein Index (IPI) human protein database (version 3.53) using the BioworksBrowser rev. 3.1 (Thermo Fisher Scientific Inc., Cambridge, MA, USA).

### Immunoprecipitation and western blotting

For immunoprecipitation, 293T cells was transfected with pCDNA3.0-GRK6-Flag construct or pCDNA3.0-GFP and harvested 48 h later. Cells were washed with ice-cold PBS and lysed in IP buffer (50 mM HEPES, pH 8.0, 250 mM NaCl, 0.5% NP-40, 10% glycerol, 2 mM EDTA, 1 mM Na_3_VO_4_, 10 *μ*g/ml aprotinin, 10 *μ*g/ml benzamidine, and 0.2 mM PMSF) for 1.5 h as described.^46^ The supernatant was incubated with anti-FLAG M2 affinity gel at 4 °C for 8 h. The beads were subsequently washed, and the proteins bound to the beads were subjected to western procedures.

The following antibodies were used, cMyc (sc-764), cMyc (pS62) (ab51156), DNA-PKcs (pT2056) (ab18192), ATM (pS1981) (ab36810), DNA-PKcs (ab70250), ATM (ab82512), *β*-actin (AC-15), GRK4-6 (A16/17), GRK6 (EPR2046(2)), FLAG epitope (SIG1-25). Anti-Flag M2 affinity gel (A2220) were from Sigma-Aldrich Co. LLC. (St. Louis, MO, USA). Blots were incubated with IRDye 800CW-conjugated or 700CW-conjugated antibody (Rockland Immunochemicals Inc., Limerick, PA, USA) and infrared fluorescence images were obtained with the Odyssey CLx infrared imaging system and quantified with Image Studio Lite Ver 5.2 (LI-COR Inc., Lincoln, NE, USA).

### Reverse Transcription and real-time polymerase chain reaction

PrimeScript RT reagent Kit (Takara Biotechnology (Dalian) Co, LTD., Dalian, China) was used for regular reverse transcription. For HSC and CLP populations, cells were sorted and subjected to reverse transcription and cDNA amplification according to Smart-seq2 method. Briefly, collected cells were subjected to reverse transcription, template switching and preamplification for 20 cycles, and validated for housekeeping gene expression. Then amplified cDNA was purified, and used for RT-PCR detection. PCR primers were designed with NCBI Primer Blast and tested for PCR band specificity and excluded for genomic DNA amplification. Real-time PCR was done on an Eppendorf Realplex 2 real-time PCR cycler using the Takara SYBR Green PCR kit. All samples were run in duplicate. Relative expression was determined using the 2^−ΔΔC^^T^ method. See Supplementary Table 1 for primers used.

### RNA sequencing and data analysis

Sample preparation was performed as described.^47, 48^ Briefly, sorted cells were individually transferred into lysis buffer with a mouth pipette. Reverse transcription reactions were directly performed on the whole-cell lysate. Poly (dT) primer was used to specifically reverse transcribe mRNA from the lysate. Terminal deoxynucleotidyl transferase was used to add a poly (A) tail to the 3′ end of the first-strand cDNA. The unincorporated primer was digested by exonuclease and 20+10 cycles of PCR was applied to amplify the cDNA. Sorted cells (13–15) from 3–4 mice were individually amplified and pooled together as cDNA libraries. Library preparation and sequencing were performed by WuXi AppTec. We obtained 20 million 100-bp PE reads for each lineage. Data analysis was performed with Genome assembly GRCm37/mm9. Data were aligned and calculated for differential expression with the Tuxedo suite.^49, 50^ Network analysis of over-represented pathways in RNA-seq by Enrichment Map. Gene sets were organized in a network, where each set was a node and edges represented gene overlap between sets. Automated network layout groups related gene sets into network clusters, indicated by grey shadows. Terms in each cluster were summarized by WordCloud.

### Trolox-equivalent antioxidant capacity measurement

Cellular antioxidant capacity was measured by commercial total antioxidant capacity assay kit with a rapid ABTS (2,2′-azino-bis(3-ethylbenzithiazoline)-6-sulfonic acid) method (Beyotime Biotechnology, Haimen, China). Briefly, Jurkat cells were collected, washed with ice-cold PBS, and homogenized with a tissue lyser. Supernatant of cell lysate were subjected to ABTS^+^ measurement at A414 nm, and BCA method was used to determine total protein level of the lysate. Total antioxidant capacity was evaluated by a Trolox (6-hydroxy-2,5,7,8-tetramethylchroman-2-carboxylic acid) standard curve and was expressed as Trolox-equivalent antioxidant capacity concentration (mM/g).

### Statistical analysis

Data from an animal was excluded if it deceased before the experiment procedure ends. Quasi experiments were performed in all tests, during which groups were masked until the end of experiment. Statistical analysis was performed with two-tailed, unpaired Student's *t*-test or two-way ANOVA followed by Bonferroni's *post poc* analysis, as indicated in figure legends. Data were presented as mean±S.E.M. We conducted a range of estimations based on alpha values of 0.05 and desired power of 0.80. Significance was denoted with asterisks (*P<0.05, **P<0.01, ***P<0.001). Plotting and statistics were done with Sigmaplot 12.5 (Systat Software Inc., San Jose, CA, USA) or R.

### Data availability

All the raw data of RNA sequencing for WT and GRK6^−/−^ HSC and CLP have been deposited in the Gene Expression Omnibus database (accession number GSE58357).
