# Supplementary material for: GRK6 regulates ROS response and maintains hematopoietic stem cell self-renewal
Source: Cell Death Dis. 2016 Nov 24;7(11):e2478–. doi: 10.1038/cddis.2016.377 (PMC5260904; doi:10.1038/cddis.2016.377)
Supplement: Supplementary Information [file cddis2016377x1.pdf]

Supplemental table

Table S1 Oligos used in realtime-PCR experiments.

Gene	Species	Forward	Reverse
Grk6	hsa	AGCGTGACTATCACAGCCTG	CTTCCGCTTGTCATCCGGG
Rpl13a	hsa	CCAAGCGGCTGCCGAAGATGG	CTTCCGGCCCAGCAGTACCTGT
Nox2	mmu	CTGAAGGGGGCCTGTATGTG	CCAAACTCTCCGCAGTCTGT
Nox4	mmu	TGTTGGGCCTAGGATTGTGT	CAGGACTGTCCGGCACATAG
Cdkn1a	mmu	TCAGGCGCAGATCCACAGCG	CGAACGCGCTCCCAGACGAA
Sod3	mmu	GAGCTCTTGGGAGAGCCTGA	GCTCCATCCAGATCTCCAGC
Nox1	mmu	CCTGATTCCTGTGTGTCGAAA	TTGGCTTCTTCTGTAGCGTTC
Gapdh	mmu	TGTGTCCGTCGTGGATCTGA	CCTGCTTCACCACCTTCTTGA
Sod1	mmu	ATGGCGATGAAAGCGGTGT	CAGTCACATTGCCCAGGTCT
Sod2	mmu	TTCTGGACAAACCTGAGCCC	GTCACGCTTGATAGCCTCCA
Grk6	mmu	TTCCCCCATATCAGCCAGTGT	CGTAGCACAGAACTCACGAAAT
Nrf2	mmu	TCTTGGAGTAAGTCGAGAAGTGT	GTTGAAACTGAGCGAAAAAGGC

Supplemental data

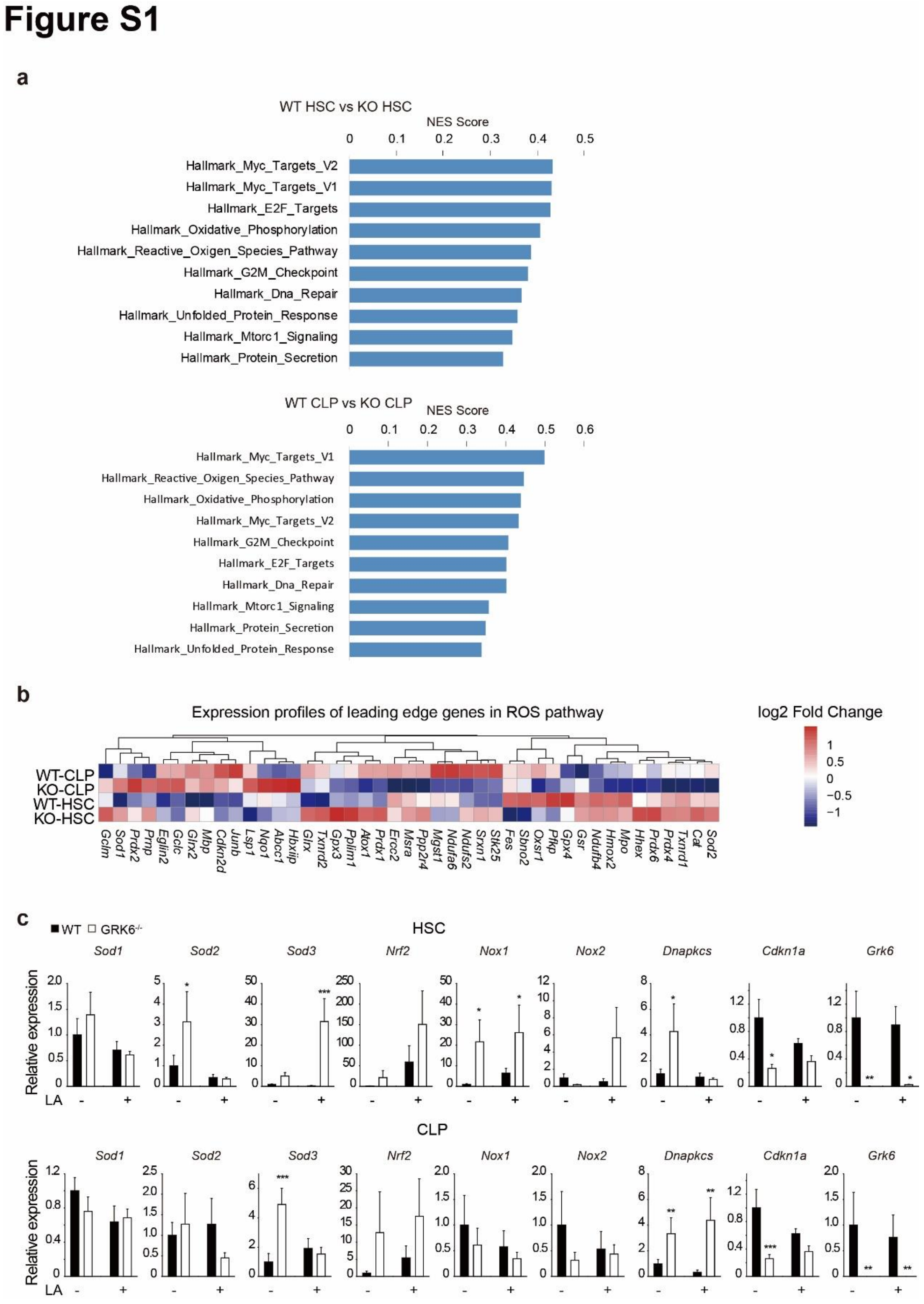

Figure S1. GRK6 ablation leads to alterations in ROS pathway. (a) Top ten significantly changed hallmark pathways in HSC and CLP caused by GRK6 ablation. (b) Expression profiles

of leading edge genes in ROS pathway were presented. (e) Real-time PCR quantification of *Sod1*, *Sod2*, *Sod3*, *Nrf2*, *Nox1*, *Nox2*, *Dna-pkcs*, *Cdkn1a*, *Grk6* (WT + Saline, WT + LA, GRK6$^{-/-}$ + Saline, GRK6$^{-/-}$ + LA, N = 5, 4, 3, 3). Two-way ANOVA with Bonferroni *post hoc;* * $P < 0.05$, ** $P < 0.01$, *** $P < 0.001$, within genotype. Data are expressed as Mean ± SEM.

Figure S2

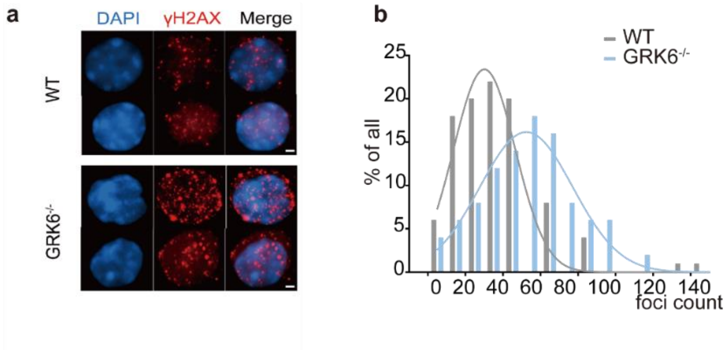

Figure S2. More γ-H2AX foci in GRK6$^{-/-}$ than WT HSC. Sorted cells were stained for intracellular γ-H2AX using phospho-specific antibody, and photographed by confocal microscopy. Foci of γ-H2AX positive dots from 100 cells were counted in each population. (a) Representative immunofluorescence graphs . Objective, Plan-Apochromat 63×/1.4. Scale Bar, 2 μm. (b) Plot of distribution. t (198) = 3.138, p = 0.002, two-tailed, unpaired Student's t-test.

Figure S3

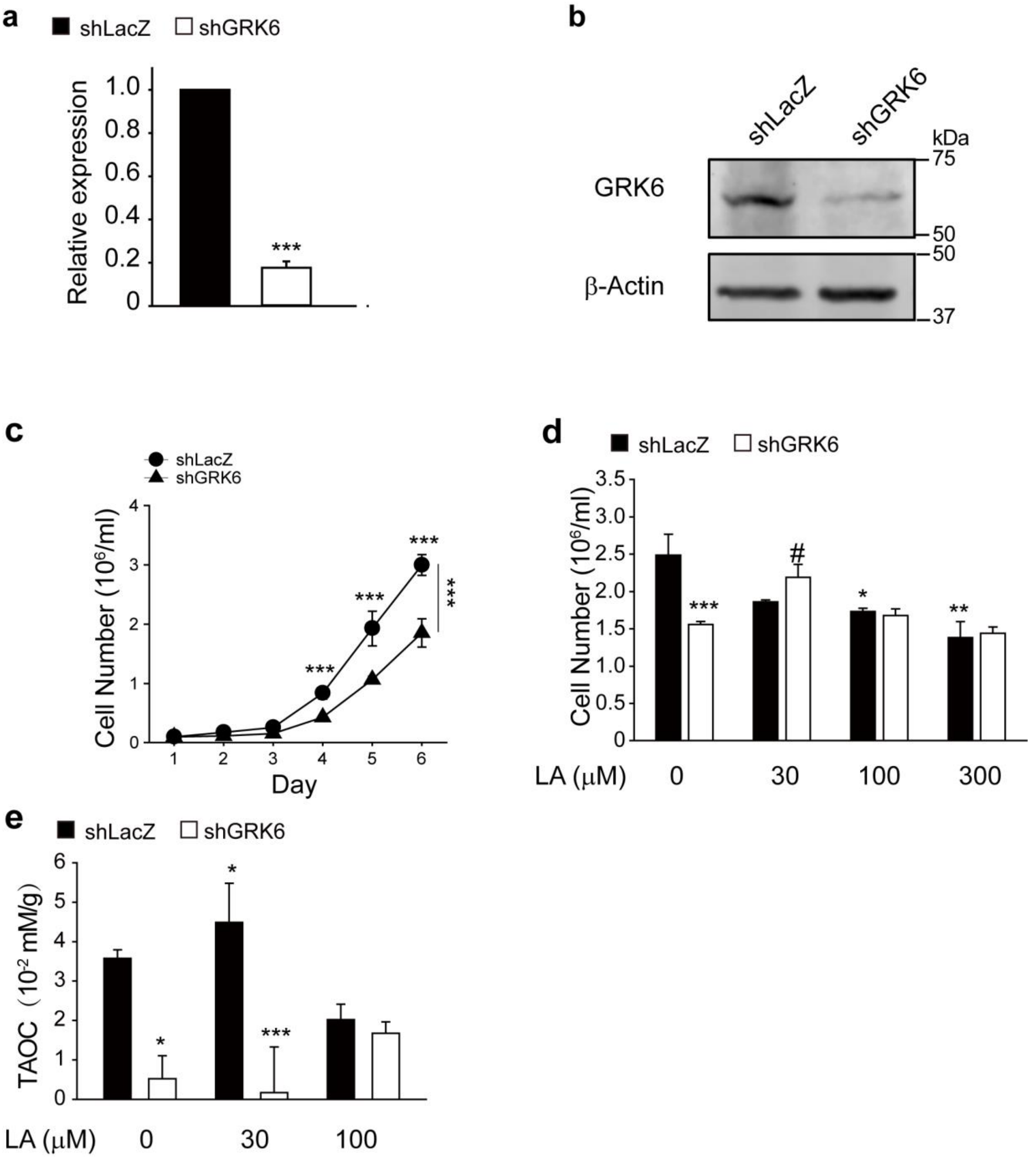

Figure S3. GRK6 knockdown leads to growth arrest and reduced antioxidative capacity. (a-b) GRK6 knockdown efficiency was tested, (a) in HEK293T by RT-PCR, and (b) in Jurkat by Western blot. (c) GRK6 knockdown lead to reduced cellular growth. Seventy two hours after lentiviral spinoculation, 2×10^5/ml Jurkat cells were cultured in RPMI 1640 supplemented with 10% FBS and cellular density was measured daily. $F_{virus\times\ day} = 7.141$, $P < 0.001$. (d) LA

improved cell growth in lentivirus-mediated GRK6 knockdown. $F_{virus\times\ dose} = 6.746$, $P = 0.004$. (e) LA was supplied to cell culture media and TAOC was measured 6 hr later. Two-way ANOVA with Bonferroni post hoc. * $P < 0.05$, *** $P < 0.001$, within genotype; # $P < 0.05$ within treatment. N = 4 each. Data are expressed as Mean ± SEM.

Figure S4

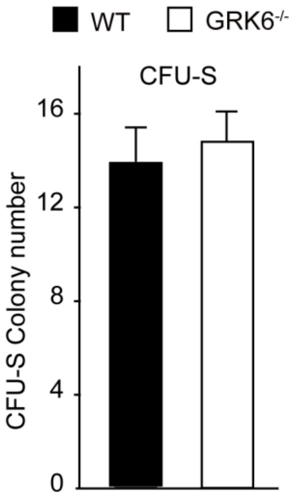

Figure S4. GRK6 is dispensable in migration and short-term reconstitution of hematopoietic progenitor cells. Spleen clonogenic (CFU-S) assay. 5×10^5 bone marrow cells from GRK6$^{-/-}$ or WT mice were retro-orbital injected into wild type recipient mice. Spleen was dissected at day 12 and enumerated for colonies (Recipients N = 5-6 each, donor N = 3 per group).
